# RECEIVING ELDERLY PRIVILEGE AND POSITIVE ATTITUDES ABOUT AGING: THE MEDIATING ROLE OF SUPPORT FROM CHILDREN

**DOI:** 10.1093/geroni/igz038.1713

**Published:** 2019-11-08

**Authors:** Lingya Zhao, Ling Xu

**Affiliations:** 1 Wuhan University, School of Political Science and Public Administration, Wuhan, Wubei province, China; 2 University of Texas at Arlington, Arlington, Texas, United States

## Abstract

Objectives. This study investigated the influence of receiving elderly privilege on positive attitudes about aging, and examined the mediating roles of support from children in such associations among Chinese older adults. Methods. Data were derived from China Longitudinal Aging Social Survey (CLASS), a nationwide social survey project of Chinese older adults aged 60 or older (N = 7,184) conducted in 2014. The positive attitudes about aging were measured by Attitudes to Aging Questionnaire. Sum scores were calculated with higher scores indicating more positive attitudes about aging. Analyses were conducted by PROCESS v3.3 for SPSS 20.0. Results. Receiving elderly privilege was significantly associated with more financial support (b = 0.303, p＜.001) and instrumental support (b =0.225, p＜.001) from children. Financial support (b = 0.100, p < .05), instrumental support (b = 0.090, p < .05), and emotional support (b = 0.405, p＜.001) received from children were significantly correlated with more positive attitudes about aging. Financial support (b = 0.030, BCa CI [0.010, 0.057]) and instrumental support (b = 0.020, BCa CI [0.000, 0.042]) from children mediated the association between receiving elderly privilege and positive attitudes about aging. Conclusion. With the rapid increase of aging population in China, it’s important to help Chinese older adults maintain high quality of life by encouraging society and family to work together. To maintain higher levels of positive attitudes about aging among Chinese older adults, social program of providing elderly privilege and support from children are both needed.

